# (*Z*)-1,3,4a-Trimethyl-5,5-diphenyl-6-oxa-1,3-diaza­bicyclo­[4.2.0]octane-2,4-dione

**DOI:** 10.1107/S1600536808006557

**Published:** 2008-04-04

**Authors:** Zhi-Cai Lin, Jing-Bo Shi, Wen-Jian Tang, Jun Li

**Affiliations:** a School of Pharmacy, Anhui Medical University, Hefei 230032, People’s Republic of China

## Abstract

The title compound, C_20_H_20_N_2_O_3_, is a head-to-tail oxetane, one of the regioisomers obtained by the the Paternó–Büchi reaction of 1,3-dimethyl­thymine with benzophenone. The oxetane ring is folded, the dihedral angle between the C—O—C and C—C—C planes being 14.4 (2)°. The dihedral angle between the two phenyl rings is 64.3 (2)°. The pyrimidine ring adopts a boat conformation. The crystal structure involves weak C—H⋯O hydrogen bonds.

## Related literature

For related literature, see: Hei *et al.* (2005 ▶); Prakash *et al.* (1997 ▶).
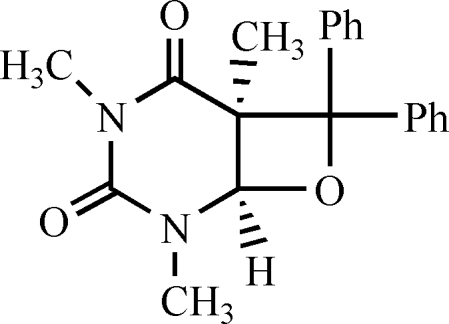


## Experimental

### 

#### Crystal data

C_20_H_20_N_2_O_3_
*M* *_r_* = 336.38
Monoclinic, 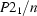

*a* = 8.1341 (7) Å
*b* = 9.1004 (13) Å
*c* = 23.485 (2) Å
β = 97.334 (2)°
*V* = 1724.2 (3) Å^3^
*Z* = 4
Mo *K*α radiation
μ = 0.09 mm^−1^
*T* = 298 (2) K
0.45 × 0.41 × 0.18 mm

#### Data collection

Bruker SMART diffractometer
Absorption correction: multi-scan (*SADABS*; Sheldrick, 1996 ▶) *T*_min_ = 0.962, *T*_max_ = 0.984
8774 measured reflections
3039 independent reflections
1818 reflections with *I* > 2σ(*I*)
*R*_int_ = 0.036

#### Refinement

*R*[*F* ^2^ > 2σ(*F* ^2^)] = 0.044
*wR*(*F* ^2^) = 0.117
*S* = 1.01
3039 reflections
226 parameters
H-atom parameters constrained
Δρ_max_ = 0.20 e Å^−3^
Δρ_min_ = −0.18 e Å^−3^

### 

Data collection: *SMART* (Siemens, 1996 ▶); cell refinement: *SAINT* (Siemens, 1996 ▶); data reduction: *SAINT*; program(s) used to solve structure: *SHELXS97* (Sheldrick, 2008 ▶); program(s) used to refine structure: *SHELXL97* (Sheldrick, 2008 ▶); molecular graphics: *SHELXTL* (Sheldrick, 2008 ▶); software used to prepare material for publication: *SHELXTL*.

## Supplementary Material

Crystal structure: contains datablocks I, global. DOI: 10.1107/S1600536808006557/wn2239sup1.cif

Structure factors: contains datablocks I. DOI: 10.1107/S1600536808006557/wn2239Isup2.hkl

Additional supplementary materials:  crystallographic information; 3D view; checkCIF report

## Figures and Tables

**Table 1:** Hydrogen-bond geometry (Å, °)

*D*—H⋯*A*	*D*—H	H⋯*A*	*D*⋯*A*	*D*—H⋯*A*
C3—H3⋯O2^i^	0.98	2.53	3.431 (3)	153
C7—H7⋯O1	0.93	2.37	2.745 (3)	104
C8—H8⋯O1^ii^	0.93	2.59	3.513 (3)	171
C13—H13⋯O1	0.93	2.41	2.752 (3)	101
C17—H17⋯O3	0.93	2.55	3.040 (3)	113
C20—H20*B*⋯O2	0.96	2.29	2.682 (4)	104

Symmetry codes: (i) 
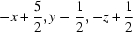
; (ii) 
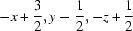
.

**Fig. 1.**
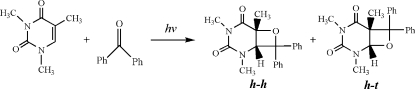
Formation of the two regioisomers of the Paternó-Büchi reaction.

**Fig. 2.**
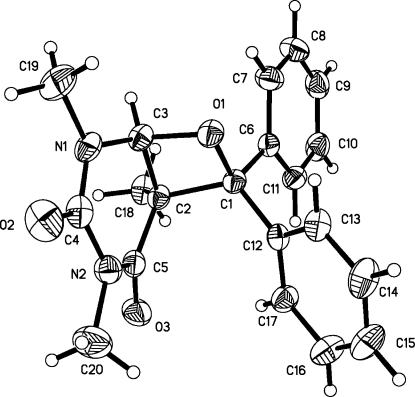
Molecular structure of the title compound, with the atomic numbering scheme, and displacement ellipsoids drawn at the 30% probability level.

## supplementary crystallographic information

### Comment

A [2 + 2] photocycloaddition (Paternò-Büchi reaction) of the 5–6 double bond of 1,3-dimethylthymine (DMT) with the carbonyl of benzophenone generates two regioisomers, head-to-head and head-to-tail oxetanes (Fig. 1). We have observed the temperature dependence of the regioselectivity (Hei *et al.*, 2005). The crystal structure of the head-to-head oxetane has already been published (Prakash *et al.*, 1997). In the present study, an X-ray crystallographic analysis of the head-to-tail oxetane has been undertaken to establish its structure and configuration.

The structure is similar to that observed in the head-to-head oxetane (Prakash *et al.*, 1997). The bond lengths and angles in the title compound are in good agreement with expected values. The oxetane ring is folded and the dihedral angle between the C1—O1—C3 and C1—C2—C3 planes is 14.4 (2)° (Fig. 2). The dihedral angle between the two phenyl rings is 64.3 (2)° and the pyrimidine ring adopts a boat conformation. The crystal structure involves weak C—H···O hydrogen bonds.

### Experimental

The title compound was prepared by first dissolving DMT (0.77 g, 5.0 mmol) and benzophenone (1.82 g, 10.0 mmol) in CH_3_CN (100 ml). The resulting solution was placed in a photochemical apparatus (Pyrex), purged with nitrogen, degassed for 30 min, and irradiated with a 300 W mercury high-pressure lamp for 10 h. The solvent was then rotary evaporated and the oxetane was purified by silica gel chromatography using a 4:1 petroleum ether/ethyl acetate solvent mixture. The fractions containing the h-t oxetane were combined, and the solvent removed by rotary evaporation. The purified oxetane was subsequently crystallized by dissolving the residue in 10 ml of ethyl acetate and adding n-hexane to the solution until it turned cloudy. Upon standing at room temperature, a colorless block appeared and was separated from the solvent by decanting.

### Refinement

All H atoms were positioned geometrically and refined using a riding model, with C—H = 0.98 Å (methine), 0.96 Å (methyl) and 0.93 Å (aromatic); *U*_iso_(H) = 1.2 *U*_eq_(C, O) or 1.5 *U*_eq_(C) for the methyl group.

### Crystal data

**Table:**

C_20_H_20_N_2_O_3_	*F*_000_ = 712
*M**_r_* = 336.38	*D*_x_ = 1.296 Mg m^−^^3^
Monoclinic, *P*2_1_/*n*	Mo *K*α radiation λ = 0.71073 Å
*a* = 8.1341 (7) Å	Cell parameters from 1952 reflections
*b* = 9.1004 (13) Å	θ = 2.4–22.1º
*c* = 23.485 (2) Å	µ = 0.09 mm^−^^1^
β = 97.334 (2)º	*T* = 298 (2) K
*V* = 1724.2 (3) Å^3^	Block, colorless
*Z* = 4	0.45 × 0.41 × 0.18 mm

### Data collection

**Table:**

Bruker SMART diffractometer	3039 independent reflections
Radiation source: fine-focus sealed tube	1818 reflections with *I* > 2σ(*I*)
Monochromator: graphite	*R*_int_ = 0.036
*T* = 298(2) K	θ_max_ = 25.0º
φ and ω scans	θ_min_ = 1.8º
Absorption correction: multi-scan(SADABS; Sheldrick, 1996)	*h* = −9→9
*T*_min_ = 0.962, *T*_max_ = 0.984	*k* = −10→10
8774 measured reflections	*l* = −24→27

### Refinement

**Table:**

Refinement on *F*^2^	Secondary atom site location: difference Fourier map
Least-squares matrix: full	Hydrogen site location: inferred from neighbouring sites
*R*[*F*^2^ > 2σ(*F*^2^)] = 0.044	H-atom parameters constrained
*wR*(*F*^2^) = 0.117	*w* = 1/[σ^2^(*F*_o_^2^) + (0.038*P*)^2^ + 0.7238*P*] where *P* = (*F*_o_^2^ + 2*F*_c_^2^)/3
*S* = 1.01	(Δ/σ)_max_ < 0.001
3039 reflections	Δρ_max_ = 0.20 e Å^−^^3^
226 parameters	Δρ_min_ = −0.18 e Å^−^^3^
Primary atom site location: structure-invariant direct methods	Extinction correction: none

### Fractional atomic coordinates and isotropic or equivalent isotropic displacement parameters (Å^2^)

**Table:**

	*x*	*y*	*z*	*U*_iso_*/*U*_eq_	
N1	1.2692 (2)	0.5663 (2)	0.21196 (8)	0.0491 (5)	
N2	1.2831 (2)	0.6885 (2)	0.12510 (8)	0.0492 (5)	
O1	0.97763 (19)	0.60800 (17)	0.19685 (6)	0.0469 (4)	
O2	1.4490 (2)	0.7527 (2)	0.20642 (8)	0.0750 (6)	
O3	1.1732 (2)	0.5833 (2)	0.04218 (7)	0.0625 (5)	
C1	0.9036 (3)	0.5849 (2)	0.13794 (8)	0.0379 (5)	
C2	1.0673 (3)	0.4991 (2)	0.12589 (9)	0.0380 (5)	
C3	1.1126 (3)	0.5076 (3)	0.19078 (9)	0.0427 (6)	
H3	1.0949	0.4127	0.2088	0.051*	
C4	1.3391 (3)	0.6743 (3)	0.18356 (11)	0.0503 (6)	
C5	1.1782 (3)	0.5906 (3)	0.09400 (10)	0.0440 (6)	
C6	0.7544 (3)	0.4834 (2)	0.13563 (9)	0.0398 (6)	
C7	0.7166 (3)	0.4145 (3)	0.18451 (11)	0.0555 (7)	
H7	0.7781	0.4358	0.2197	0.067*	
C8	0.5890 (4)	0.3147 (3)	0.18183 (13)	0.0654 (8)	
H8	0.5646	0.2695	0.2153	0.079*	
C9	0.4977 (3)	0.2812 (3)	0.13056 (13)	0.0617 (8)	
H9	0.4128	0.2124	0.1289	0.074*	
C10	0.5325 (3)	0.3502 (3)	0.08148 (12)	0.0580 (7)	
H10	0.4701	0.3289	0.0465	0.070*	
C11	0.6592 (3)	0.4506 (3)	0.08398 (10)	0.0481 (6)	
H11	0.6815	0.4971	0.0506	0.058*	
C12	0.8626 (3)	0.7299 (2)	0.10882 (9)	0.0385 (6)	
C13	0.8285 (3)	0.8494 (3)	0.14167 (11)	0.0505 (6)	
H13	0.8336	0.8392	0.1813	0.061*	
C14	0.7872 (4)	0.9835 (3)	0.11655 (14)	0.0673 (8)	
H14	0.7645	1.0629	0.1392	0.081*	
C15	0.7794 (4)	0.9999 (3)	0.05856 (14)	0.0727 (9)	
H15	0.7523	1.0906	0.0417	0.087*	
C16	0.8116 (4)	0.8830 (3)	0.02531 (12)	0.0666 (8)	
H16	0.8061	0.8942	−0.0142	0.080*	
C17	0.8520 (3)	0.7485 (3)	0.05001 (10)	0.0504 (6)	
H17	0.8724	0.6693	0.0269	0.060*	
C18	1.0451 (3)	0.3473 (3)	0.09951 (10)	0.0503 (6)	
H18A	0.9886	0.3552	0.0612	0.075*	
H18B	0.9809	0.2877	0.1222	0.075*	
H18C	1.1517	0.3029	0.0985	0.075*	
C19	1.3263 (4)	0.5493 (3)	0.27297 (11)	0.0783 (9)	
H19A	1.4432	0.5683	0.2799	0.117*	
H19B	1.3046	0.4508	0.2847	0.117*	
H19C	1.2686	0.6175	0.2945	0.117*	
C20	1.3705 (4)	0.7944 (3)	0.09317 (13)	0.0803 (10)	
H20A	1.4539	0.7445	0.0751	0.121*	
H20B	1.4218	0.8676	0.1190	0.121*	
H20C	1.2931	0.8408	0.0644	0.121*	

### Atomic displacement parameters (Å^2^)

**Table:**

	*U*^11^	*U*^22^	*U*^33^	*U*^12^	*U*^13^	*U*^23^
N1	0.0490 (13)	0.0549 (13)	0.0392 (12)	0.0034 (11)	−0.0110 (9)	−0.0031 (10)
N2	0.0448 (12)	0.0524 (13)	0.0499 (13)	−0.0048 (10)	0.0035 (10)	−0.0005 (10)
O1	0.0509 (10)	0.0554 (11)	0.0329 (9)	0.0088 (8)	−0.0009 (7)	−0.0072 (7)
O2	0.0666 (13)	0.0819 (14)	0.0730 (13)	−0.0225 (12)	−0.0044 (11)	−0.0256 (11)
O3	0.0560 (12)	0.0921 (14)	0.0402 (11)	−0.0015 (10)	0.0092 (8)	−0.0015 (9)
C1	0.0403 (14)	0.0433 (14)	0.0290 (12)	0.0050 (11)	0.0004 (10)	−0.0041 (10)
C2	0.0387 (13)	0.0403 (13)	0.0336 (12)	0.0042 (11)	−0.0008 (10)	−0.0043 (10)
C3	0.0467 (15)	0.0395 (14)	0.0402 (14)	0.0043 (12)	−0.0011 (11)	−0.0030 (11)
C4	0.0450 (15)	0.0526 (17)	0.0519 (16)	0.0041 (13)	0.0012 (13)	−0.0122 (13)
C5	0.0377 (14)	0.0522 (16)	0.0414 (15)	0.0069 (12)	0.0024 (11)	−0.0021 (12)
C6	0.0379 (13)	0.0414 (14)	0.0405 (14)	0.0063 (11)	0.0067 (11)	0.0006 (11)
C7	0.0573 (17)	0.0665 (18)	0.0442 (15)	−0.0029 (15)	0.0119 (12)	0.0020 (13)
C8	0.0656 (19)	0.070 (2)	0.065 (2)	−0.0033 (16)	0.0253 (16)	0.0127 (15)
C9	0.0438 (16)	0.0573 (18)	0.085 (2)	−0.0021 (14)	0.0134 (15)	0.0074 (16)
C10	0.0402 (15)	0.0623 (18)	0.0680 (19)	−0.0026 (14)	−0.0062 (13)	0.0037 (15)
C11	0.0447 (15)	0.0519 (16)	0.0467 (15)	−0.0002 (13)	0.0015 (12)	0.0076 (12)
C12	0.0324 (13)	0.0391 (14)	0.0434 (14)	0.0010 (10)	0.0027 (10)	−0.0035 (11)
C13	0.0477 (15)	0.0505 (16)	0.0512 (15)	0.0059 (13)	−0.0016 (12)	−0.0098 (13)
C14	0.073 (2)	0.0418 (16)	0.083 (2)	0.0119 (14)	−0.0031 (16)	−0.0150 (15)
C15	0.087 (2)	0.0434 (17)	0.083 (2)	0.0064 (15)	−0.0078 (18)	0.0103 (16)
C16	0.089 (2)	0.0519 (18)	0.0564 (17)	0.0058 (16)	−0.0001 (15)	0.0085 (14)
C17	0.0587 (17)	0.0438 (15)	0.0483 (16)	0.0068 (13)	0.0059 (12)	0.0010 (12)
C18	0.0462 (15)	0.0501 (15)	0.0524 (15)	0.0065 (12)	−0.0026 (12)	−0.0129 (12)
C19	0.082 (2)	0.095 (2)	0.0493 (17)	−0.0024 (19)	−0.0221 (15)	0.0001 (16)
C20	0.078 (2)	0.082 (2)	0.082 (2)	−0.0240 (18)	0.0150 (18)	0.0105 (17)

### Geometric parameters (Å, °)

**Table:**

N1—C4	1.353 (3)	C9—H9	0.9300
N1—C3	1.412 (3)	C10—C11	1.373 (3)
N1—C19	1.457 (3)	C10—H10	0.9300
N2—C5	1.377 (3)	C11—H11	0.9300
N2—C4	1.396 (3)	C12—C13	1.381 (3)
N2—C20	1.460 (3)	C12—C17	1.383 (3)
O1—C3	1.450 (3)	C13—C14	1.379 (4)
O1—C1	1.452 (2)	C13—H13	0.9300
O2—C4	1.214 (3)	C14—C15	1.364 (4)
O3—C5	1.214 (3)	C14—H14	0.9300
C1—C12	1.504 (3)	C15—C16	1.365 (4)
C1—C6	1.521 (3)	C15—H15	0.9300
C1—C2	1.600 (3)	C16—C17	1.376 (3)
C2—C5	1.497 (3)	C16—H16	0.9300
C2—C18	1.515 (3)	C17—H17	0.9300
C2—C3	1.523 (3)	C18—H18A	0.9600
C3—H3	0.9800	C18—H18B	0.9600
C6—C7	1.376 (3)	C18—H18C	0.9600
C6—C11	1.386 (3)	C19—H19A	0.9600
C7—C8	1.375 (4)	C19—H19B	0.9600
C7—H7	0.9300	C19—H19C	0.9600
C8—C9	1.366 (4)	C20—H20A	0.9600
C8—H8	0.9300	C20—H20B	0.9600
C9—C10	1.373 (4)	C20—H20C	0.9600
			
C4—N1—C3	121.2 (2)	C11—C10—C9	120.2 (3)
C4—N1—C19	117.4 (2)	C11—C10—H10	119.9
C3—N1—C19	117.8 (2)	C9—C10—H10	119.9
C5—N2—C4	124.3 (2)	C10—C11—C6	120.9 (2)
C5—N2—C20	117.6 (2)	C10—C11—H11	119.6
C4—N2—C20	116.6 (2)	C6—C11—H11	119.6
C3—O1—C1	92.41 (14)	C13—C12—C17	118.0 (2)
O1—C1—C12	110.28 (17)	C13—C12—C1	119.0 (2)
O1—C1—C6	110.74 (17)	C17—C12—C1	122.9 (2)
C12—C1—C6	112.78 (18)	C14—C13—C12	120.9 (2)
O1—C1—C2	89.20 (14)	C14—C13—H13	119.6
C12—C1—C2	119.28 (18)	C12—C13—H13	119.6
C6—C1—C2	112.08 (17)	C15—C14—C13	120.1 (3)
C5—C2—C18	110.54 (19)	C15—C14—H14	119.9
C5—C2—C3	112.83 (19)	C13—C14—H14	119.9
C18—C2—C3	117.16 (19)	C14—C15—C16	119.9 (3)
C5—C2—C1	112.63 (18)	C14—C15—H15	120.1
C18—C2—C1	117.30 (18)	C16—C15—H15	120.1
C3—C2—C1	84.20 (15)	C15—C16—C17	120.3 (3)
N1—C3—O1	112.77 (18)	C15—C16—H16	119.8
N1—C3—C2	117.63 (19)	C17—C16—H16	119.8
O1—C3—C2	92.34 (15)	C16—C17—C12	120.7 (2)
N1—C3—H3	110.9	C16—C17—H17	119.6
O1—C3—H3	110.9	C12—C17—H17	119.6
C2—C3—H3	110.9	C2—C18—H18A	109.5
O2—C4—N1	122.7 (2)	C2—C18—H18B	109.5
O2—C4—N2	120.6 (3)	H18A—C18—H18B	109.5
N1—C4—N2	116.6 (2)	C2—C18—H18C	109.5
O3—C5—N2	120.4 (2)	H18A—C18—H18C	109.5
O3—C5—C2	121.7 (2)	H18B—C18—H18C	109.5
N2—C5—C2	117.8 (2)	N1—C19—H19A	109.5
C7—C6—C11	118.2 (2)	N1—C19—H19B	109.5
C7—C6—C1	120.6 (2)	H19A—C19—H19B	109.5
C11—C6—C1	121.07 (19)	N1—C19—H19C	109.5
C8—C7—C6	120.7 (2)	H19A—C19—H19C	109.5
C8—C7—H7	119.7	H19B—C19—H19C	109.5
C6—C7—H7	119.7	N2—C20—H20A	109.5
C9—C8—C7	120.7 (3)	N2—C20—H20B	109.5
C9—C8—H8	119.7	H20A—C20—H20B	109.5
C7—C8—H8	119.7	N2—C20—H20C	109.5
C8—C9—C10	119.4 (3)	H20A—C20—H20C	109.5
C8—C9—H9	120.3	H20B—C20—H20C	109.5
C10—C9—H9	120.3		
			
C3—O1—C1—C12	−131.48 (18)	C20—N2—C5—C2	−170.0 (2)
C3—O1—C1—C6	102.98 (19)	C18—C2—C5—O3	37.6 (3)
C3—O1—C1—C2	−10.39 (16)	C3—C2—C5—O3	170.9 (2)
O1—C1—C2—C5	−102.41 (19)	C1—C2—C5—O3	−95.8 (3)
C12—C1—C2—C5	10.5 (3)	C18—C2—C5—N2	−144.8 (2)
C6—C1—C2—C5	145.47 (19)	C3—C2—C5—N2	−11.4 (3)
O1—C1—C2—C18	127.57 (19)	C1—C2—C5—N2	81.9 (2)
C12—C1—C2—C18	−119.5 (2)	O1—C1—C6—C7	−6.9 (3)
C6—C1—C2—C18	15.5 (3)	C12—C1—C6—C7	−131.1 (2)
O1—C1—C2—C3	9.93 (15)	C2—C1—C6—C7	91.0 (2)
C12—C1—C2—C3	122.9 (2)	O1—C1—C6—C11	177.12 (19)
C6—C1—C2—C3	−102.19 (18)	C12—C1—C6—C11	53.0 (3)
C4—N1—C3—O1	−72.4 (3)	C2—C1—C6—C11	−85.0 (2)
C19—N1—C3—O1	85.8 (3)	C11—C6—C7—C8	0.7 (4)
C4—N1—C3—C2	33.3 (3)	C1—C6—C7—C8	−175.3 (2)
C19—N1—C3—C2	−168.6 (2)	C6—C7—C8—C9	0.3 (4)
C1—O1—C3—N1	132.30 (19)	C7—C8—C9—C10	−1.1 (4)
C1—O1—C3—C2	10.93 (17)	C8—C9—C10—C11	0.7 (4)
C5—C2—C3—N1	−15.1 (3)	C9—C10—C11—C6	0.4 (4)
C18—C2—C3—N1	115.0 (2)	C7—C6—C11—C10	−1.1 (3)
C1—C2—C3—N1	−127.2 (2)	C1—C6—C11—C10	174.9 (2)
C5—C2—C3—O1	102.19 (19)	O1—C1—C12—C13	−27.7 (3)
C18—C2—C3—O1	−127.7 (2)	C6—C1—C12—C13	96.7 (2)
C1—C2—C3—O1	−9.95 (15)	C2—C1—C12—C13	−128.7 (2)
C3—N1—C4—O2	160.5 (2)	O1—C1—C12—C17	154.5 (2)
C19—N1—C4—O2	2.3 (4)	C6—C1—C12—C17	−81.1 (3)
C3—N1—C4—N2	−22.0 (3)	C2—C1—C12—C17	53.5 (3)
C19—N1—C4—N2	179.8 (2)	C17—C12—C13—C14	−0.8 (4)
C5—N2—C4—O2	169.6 (2)	C1—C12—C13—C14	−178.7 (2)
C20—N2—C4—O2	3.8 (3)	C12—C13—C14—C15	0.0 (4)
C5—N2—C4—N1	−7.9 (3)	C13—C14—C15—C16	0.5 (5)
C20—N2—C4—N1	−173.7 (2)	C14—C15—C16—C17	−0.1 (5)
C4—N2—C5—O3	−157.9 (2)	C15—C16—C17—C12	−0.7 (4)
C20—N2—C5—O3	7.7 (3)	C13—C12—C17—C16	1.2 (4)
C4—N2—C5—C2	24.4 (3)	C1—C12—C17—C16	179.0 (2)

### Hydrogen-bond geometry (Å, °)

**Table:**

*D*—H···*A*	*D*—H	H···*A*	*D*···*A*	*D*—H···*A*
C3—H3···O2^i^	0.98	2.53	3.431 (3)	153
C7—H7···O1	0.93	2.37	2.745 (3)	104
C8—H8···O1^ii^	0.93	2.59	3.513 (3)	171
C13—H13···O1	0.93	2.41	2.752 (3)	101
C17—H17···O3	0.93	2.55	3.040 (3)	113
C20—H20B···O2	0.96	2.29	2.682 (4)	104

Symmetry codes: (i) −*x*+5/2, *y*−1/2, −*z*+1/2; (ii) −*x*+3/2, *y*−1/2, −*z*+1/2.
